# DNA Damage in Diabetes: From Molecular Mechanisms to Clinical Implications

**DOI:** 10.3390/ijms27219576

**Published:** 2026-10-26

**Authors:** Fiammetta Vernì, Chiara Angioli, Angelo Ferriero, Beatrice Agostini

**Affiliations:** Department of Biology and Biotechnology “Charles Darwin”, Sapienza University, 00185 Rome, Italy; chiara.angioli@uniroma1.it (C.A.); ferriero.1913456@studenti.uniroma1.it (A.F.); agostini.1917634@studenti.uniroma1.it (B.A.)

**Keywords:** diabetes, DNA damage, ROS, AGEs, DNA repair

## Abstract

A growing body of evidence indicates that diabetes is associated with pronounced DNA damage, ultimately leading to genomic instability. Chronic hyperglycemia induces the production of reactive oxygen and nitrogen species (ROS and RNS), which trigger a cascade of oxidative stress through multiple pathways. These reactive species attack DNA, causing mutations as well as single- and double-strand breaks. Hyperglycemia also promotes the non-enzymatic glycation of DNA causing the formation of advanced glycation end products (AGEs) adducts. Binding of AGEs to the receptors for AGEs (RAGEs) stimulates additional ROS production, thereby amplifying oxidative stress. This state of genomic instability is further promoted by a decline in antioxidant defenses, which allows ROS-induced damage to accumulate, and is sustained by compromised DNA repair mechanisms. Accumulating DNA damage contributes to the development of diabetic micro- and macrovascular complications by triggering destructive mechanisms such as apoptosis, fibrosis, ferroptosis, and pyroptosis that compromise organ function. Additionally, persistent DNA damage elevates the risk of diabetes-associated malignancies and compromises male reproductive health. This review synthesizes current insights into the molecular mechanisms driving hyperglycemia-induced genomic damage and highlights its broad clinical implications. Understanding the multifaceted impact of DNA damage in diabetes provides a comprehensive framework for its role in disease progression and associated comorbidities.

## 1. Introduction

Diabetes mellitus is a multifactorial disease characterized by chronic hyperglycemia resulting from defects in insulin secretion, insulin action, or both. Type 1 diabetes (T1D) is primarily an autoimmune condition driven by the immune-mediated destruction of pancreatic beta cells, leading to insulin deficiency. Type 2 diabetes (T2D)—which accounts for over 90 percent of all cases—is characterized by peripheral insulin resistance coupled with progressive secretory dysfunction, wherein target tissues fail to respond adequately to physiological insulin levels [1]. Both forms share a complex, multifactorial etiology driven by an intricate interplay between genetic predisposition and environmental triggers [2]. Less common forms of diabetes include gestational diabetes (GDM), which develops during pregnancy due to temporary glucose intolerance and usually resolves postpartum; maturity-onset diabetes of the young (MODY) caused by single-gene mutations; neonatal diabetes (NDM) and latent autoimmune diabetes in adults (LADA) [2].

According to the World Health Organization (WHO), the global prevalence of diabetes has progressively increased over recent decades, with approximately 590 million individuals currently living with the condition. This number is projected to exceed 780 million by 2045 [3]. Therefore, diabetes imposes a significant economic burden on individuals and society. Growing evidence pinpoints DNA damage as an important feature and emerging hallmark of diabetes [4]. Hyperglycemia induces DNA damage primarily through reactive oxygen species (ROS), which oxidize bases and attack the DNA backbone, leading to double-strand breaks (DSBs). Additionally, direct DNA glycation forms advanced glycation end-products (AGEs) that can cause mutations or lead to DSB formation via replication fork collapse. Furthermore, the interaction between AGEs and their receptor (RAGE) triggers additional ROS production, exacerbating DNA damage [5]. Under physiological conditions, oxidative stress is tightly controlled by endogenous antioxidant defenses, including key antioxidant enzymes like superoxide dismutase (SOD) and catalase (CAT), alongside essential micronutrients such as vitamins E, C, and B6 [6]. When DNA lesions occur, they are recognized and managed by the DNA damage response (DDR) and DNA repair machinery. The DDR relies on the master regulatory kinases ataxia-telangiectasia mutated (ATM) and ATM- and Rad3-Related (ATR) to initiate signaling cascades that coordinate DNA repair, cell cycle arrest, and chromatin remodeling [7]. The major DNA repair pathways include base excision repair (BER), which targets small, non-helix-distorting base modifications arising from oxidation, alkylation, or deamination; nucleotide excision repair (NER), which focuses on bulky DNA adducts and helix-distorting lesions; mismatch repair (MMR), which corrects replication errors and insertion–deletion loops; and homologous recombination (HR) and non-homologous end joining (NHEJ), which are the primary pathways involved in DSB repair [8].

However, DDR signaling, specific DNA repair pathways, and antioxidant systems are all compromised in the diabetic milieu, leading to the accumulation of unresolved genomic lesions that contribute to secondary pathologies. Diabetes is notoriously complicated by microvascular disorders such as nephropathy, retinopathy, and neuropathy, which profoundly impair quality of life and represent leading causes of end-stage renal disease, adult blindness, and non-traumatic lower-limb amputations worldwide. Additionally, diabetes significantly increases the risk of macrovascular diseases, including cardiovascular disease, atherosclerosis, and peripheral arterial disease [9]. Growing evidence indicates that persistent DNA damage plays a crucial role in promoting cell death via apoptosis, ferroptosis and pyroptosis. Furthermore, it triggers fibrosis that ultimately compromises organ function. In large arteries, these mechanisms impair vascular smooth muscle and endothelial cell function, thereby accelerating plaque necrosis and thrombosis [10,11]. When left unrepaired or improperly repaired, persistent DSBs can also lead to chromosomal aberrations, including deletions, duplications, amplifications, inversions, and translocations, which disrupt genomic stability and facilitate malignant transformation. Accordingly, it is well-established that patients with diabetes face a heightened risk of developing cancer, driven by the cumulative burden of chronic oxidative stress, compromised repair capacity, and persistent genomic instability [12,13]. Finally, DNA damage is increasingly recognized as a key contributor to diabetes-associated male infertility via sperm DNA fragmentation [14].

This review aims to synthesize current mechanistic evidence linking diabetes to genomic instability, focusing on the molecular pathways through which DNA damage drives diabetic complications, cancer risk, and male infertility, addressing current translational challenges in the field.

## 2. Sources of Genotoxic Stress in Diabetes

**Figure 1:**
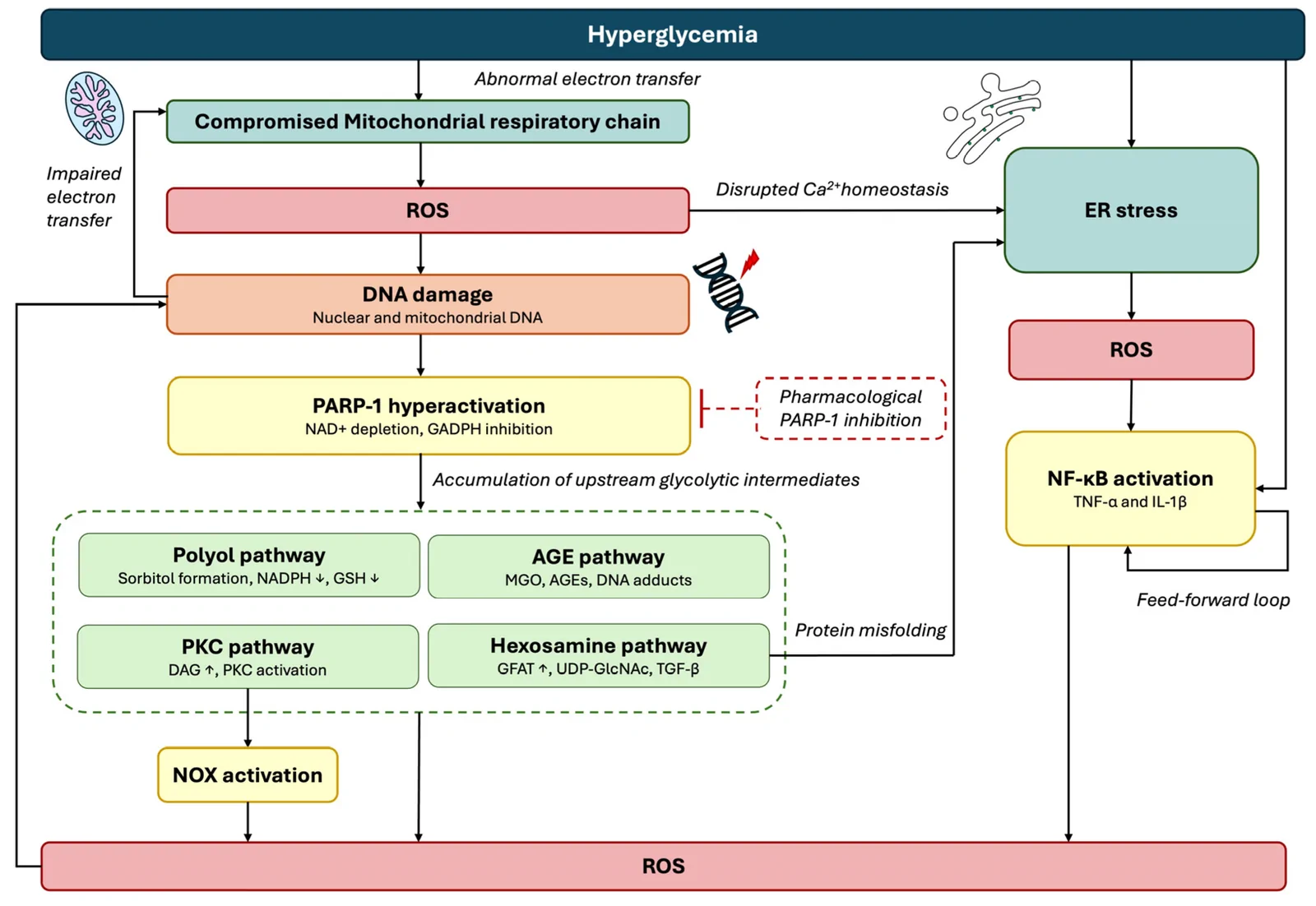
Hyperglycemia-driven ROS generation and interconnected signaling cascades. Hyperglycemia leads to increased production of reactive oxygen species (ROS) in mitochondria, which in turn generate more ROS through downstream signaling cascades. Initial ROS production induces nuclear and mitochondrial DNA damage, further impairing mitochondrial electron transfer. DNA damage leads to poly (ADP-ribose) polymerase-1 (PARP-1) activation and glyceraldehyde 3-phosphate dehydrogenase (GAPDH) inhibition (T-bar arrow), which subsequently triggers the polyol pathway, the hexosamine pathway, protein kinase C (PKC) activation, and AGE (MGO) formation. Activated PKC then stimulates NADPH oxidase (NOX) to produce additional ROS, establishing a self-amplifying loop. In parallel, hyperglycemia and endoplasmic reticulum (ER) stress drive NF-κB activation and inflammatory cytokine production, which similarly feed back to generate further ROS. In addition to hyperglycemia, ER stress is driven by both ROS-mediated disruption of Ca^2+^ homeostasis and by hexosamine pathway-induced protein misfolding. MGO = methylglyoxal. Upward (↑) and downward (↓) arrows indicate an increase and a decrease, respectively.

Hyperglycemia is a primary driver of genotoxic stress, triggering pathways that converge on DNA damage. Specifically, it impairs mitochondrial electron transport causing ROS overproduction that generates secondary ROS through alternative pathways of glucose utilization. Additionally, further ROS are generated as a consequence of inflammation and endoplasmic reticulum (ER) stress (Figure 1). Hyperglycemia also promotes AGE formation via non-enzymatic glycation reactions, which contribute to DNA damage and exacerbate oxidative stress. It also attenuates antioxidant defenses and impairs DNA repair mechanisms, increasing and perpetuating persistent genomic damage.

### 2.1. ROS

#### 2.1.1. Mitochondrial ROS Generation and Downstream Metabolic Amplification

ROS represent highly reactive molecular forms of oxygen such as superoxide anion radical, hydrogen peroxide, singlet oxygen, and hydroxyl radical. They originate primarily as byproducts of standard oxygen metabolism within mitochondria. While baseline amounts of these molecules naturally act as essential physiological regulators driving normal cell differentiation and proliferation, oxidative stress arises when ROS production exceeds the clearing capacity of the cellular antioxidant defenses [15].

In T1D and T2D, prolonged hyperglycemic stimulation leads to abnormal electron transfer within the mitochondrial respiratory chain, resulting in an excessive production of superoxide anion radical ($O_{2}^{\cdot -}$), which induces the initial wave of intracellular oxidative stress [16,17]. This excessive superoxide production inflicts immediate structural damage on both nuclear and mitochondrial DNA (mtDNA), further compromising mitochondrial electron transport and exacerbating baseline ROS production in a self-sustaining loop [18] (Figure 1). Superoxide can also react with nitric oxide (NO), the primary RNS, thus generating peroxynitrite (${\mathrm{ONOO}}^{-}$), a highly genotoxic compound capable of damaging DNA [19].

It has been proposed that extensive nuclear DNA damage induced by mitochondrial ROS and peroxynitrite triggers the hyperactivation of the DNA repair enzyme poly (ADP-ribose) polymerase-1 (PARP-1) [20]. Once hyperactivated, PARP-1 rapidly depletes its substrate, NAD^+^, slowing the rate of glycolysis, electron transport, and ATP formation. In addition, it catalyzes the ribosylation and functional inhibition of glyceraldehyde 3-phosphate dehydrogenase (GAPDH), the key glycolytic enzyme that converts glyceraldehyde 3-phosphate (GAP) into 1,3-bisphosphoglycerate (1,3-BPG). This GAPDH inhibition acts as a metabolic bottleneck that arrests normal glycolytic flux, causing upstream triose phosphate intermediates to accumulate and simultaneously overflow into four distinct parallel pathogenic pathways: the polyol pathway, the protein kinase C (PKC) pathway, the hexosamine biosynthetic pathway (HBP) and the AGE pathway (Figure 1). In the polyol pathway, excess glucose is converted to sorbitol by aldose reductase (AR), consuming NADPH. Because NADPH is an indispensable cofactor for glutathione reductase, its continuous depletion severely compromises intracellular reduced glutathione (GSH) pools, impairing endogenous antioxidant defenses and triggering downstream secondary oxidative stress [21].

Hyperglycemia significantly increases intracellular diacylglycerol (DAG) content, activating the protein kinase C (PKC) pathway. Upon activation, PKC upregulates ROS-generating enzymes such as NADPH oxidases (NOXs), directly exacerbating the oxidative microenvironment [22]. NOX family members are transmembrane proteins that catalyze the reduction of oxygen to generate ROS, such as superoxide and hydrogen peroxide, in association with NADPH oxidation; increased NOX activity has been associated with diabetes and its complications [23,24].

In the hexosamine biosynthetic pathway (HBP), increased glucose flux through glutamine-fructose-6-phosphate amidotransferase (GFAT)—the rate-limiting enzyme of this pathway—leads to excessive synthesis of UDP-N-acetylglucosamine (UDP-GlcNAc). This drives hyper-*O*-GlcNAcylation of key transcription factors, sustaining the expression of pro-inflammatory cytokines such as TGF-*β* that feed back into cellular oxidative damage. Additionally, HBP hyperactivation increases endoplasmic reticulum (ER) stress by promoting protein misfolding, thereby triggering the unfolded protein response and further ROS production [25].

Finally, accumulated triose phosphates spontaneously break down into reactive dicarbonyl compounds, predominantly methylglyoxal (MGO). Due to its electrophilic nature, MGO rapidly modifies cellular proteins, lipids, and DNA, acting as the primary precursor for AGE formation and bulky DNA adducts [26].

Preclinical findings indicate that pharmacological inhibition of PARP-1 can simultaneously block these four pathways, thereby halting the self-sustaining cycle of ROS amplification [27] (Figure 1).

ROS generation is further amplified by defects in the Mitochondrial Quality Control (MQC) network. In diabetes, suppressed mitochondrial biogenesis and fusion—combined with excessive fission and impaired mitophagy—lead to the retention of dysfunctional mitochondria, thereby adding a parallel layer of organelle-driven oxidative stress [28].

#### 2.1.2. Extra-Mitochondrial ROS Sources

##### Inflammation

Genotoxic stress is heavily amplified by systemic and local inflammatory pathways. High glucose concentrations directly stimulate the nuclear translocation of nuclear factor kappa-light-chain-enhancer of activated B cells (NF-κB), which is involved in the transcription of major pro-inflammatory cytokines, including Tumor necrosis factor-α (TNF-α) and Interleukin-1β (IL-1β). These molecules promote ROS formation by activating various cellular pathways including those leading to NOX activation and mitochondrial dysfunction. Moreover, binding back to surface receptors, they persistently reactivate NF-κB signaling through a continuous feed-forward loop [29,30] (Figure 1).

##### Endoplasmic Reticulum (ER) Stress

Endoplasmic reticulum (ER) stress represents an additional mechanism of ROS generation in the pathogenesis of diabetes. Hyperglycemia and initial mitochondrial ROS accumulation disrupt calcium homeostasis and protein folding within the ER lumen, thereby inducing acute ER stress. Although the unfolded protein response (UPR) is initially engaged to restore protein folding function, unresolved or chronic ER stress ultimately activates pro-apoptotic signaling pathways [31,32]. Protein folding induces ROS production through two primary pathways: direct electron transfer from substrate thiols to molecular oxygen, and depletion of cellular GSH reserves consumed during the reduction of unstable or mispaired disulfide bonds [33]. Consequently, in diabetes, the accumulation of misfolded proteins drives a profound increase in ROS levels, overwhelming cellular defenses. These elevated ROS subsequently activate pro-inflammatory signaling cascades, notably the NF-κB pathway, which induces pro-inflammatory cytokine expression in endothelial cells, thus stimulating further ROS production [34] (Figure 1). Consistently, transcriptomic and proteomic data demonstrate widespread translational dysregulation and ER stress in beta cells and islets in both T1D and T2D [35].

#### 2.1.3. ROS-Mediated DNA Damage

ROS accumulation induces oxidative DNA damage, a major driver of mutagenesis. Due to its low redox potential, guanine is particularly susceptible to oxidation, leading to the formation of 8-oxo-7,8-dihydro-2′-deoxyguanosine (8-oxodG), also commonly referred to as 8-hydroxy-2′-deoxyguanosine (8-OHdG). Because of its stability, the quantification of 8-oxodG/8-OHdG levels in biological samples, including blood, urine, and tissues, serves as a biomarker for assessing the extent of oxidative DNA damage. 8-oxodG is excised by 8-oxoguanine DNA glycosylase (OGG1) through the BER pathway [36]. As a pre-mutagenic lesion, 8-oxodG can mispair with adenine instead of cytosine during DNA replication, leading to a G:A mismatch. A subsequent round of replication converts this oxidative damage into a permanent G:C to T:A mutation. To prevent this, the mutY DNA glycosylase (MUTYH) recognizes and removes the misincorporated adenine opposite 8-oxodG [37]. Additionally, persistent 8-oxodG lesions can induce replication fork stalling and collapse, ultimately leading to DSBs [38]. ROS can also generate DSBs directly through oxidative cleavage of the deoxyribose backbone of DNA. If left unrepaired or misrepaired, these DSBs can cause cell death and chromosomal instability [39].

### 2.2. The Advanced Glycation End-Products (AGEs)

**Figure 2:**
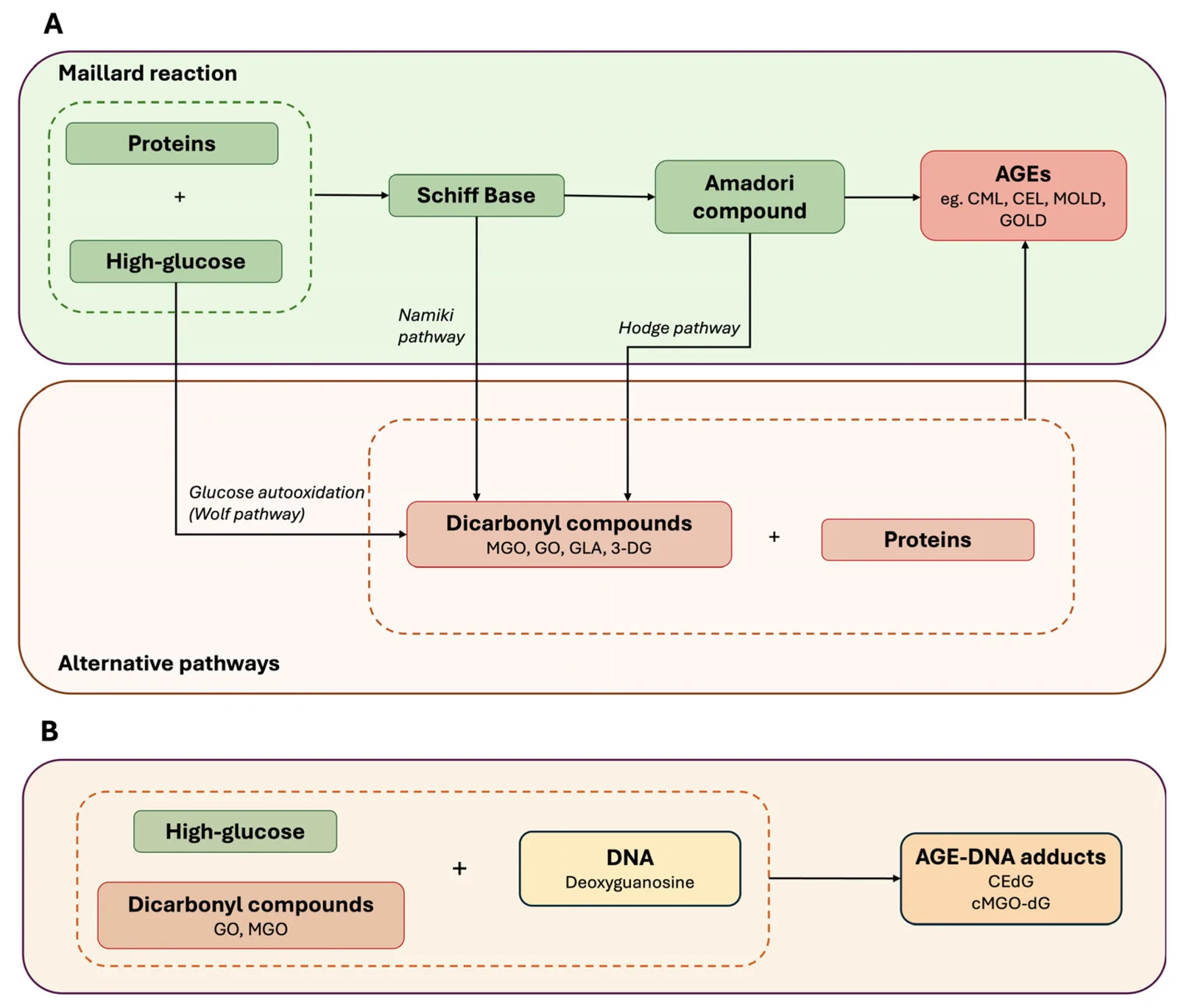
Pathways of AGE formation. High glucose levels drive Advanced Glycation End-product (AGE) formation through classical non-enzymatic glycation and alternative dicarbonyl pathways. (**A**) In the classical Maillard reaction, high glucose reacts with proteins to form Schiff bases, which rearrange into Amadori compounds that ultimately yield AGEs (including CML, CEL, MOLD, and GOLD). Concurrently, reactive dicarbonyl compounds (MGO, GO, GLA, 3-DG) are generated via three secondary routes: direct glucose auto-oxidation (Wolff pathway), degradation of Schiff bases (Namiki pathway), and breakdown of Amadori products (Hodge pathway). These intermediate dicarbonyls rapidly react with proteins, further accelerating AGE accumulation. MGO = Methylglyoxal; GO = Glyoxal; GLA = glycolaldehyde; 3-DG = 3-Deoxyglucosone; CML = $N^{\epsilon }$-(carboxymethyl)lysine; CEL = $N^{\epsilon }$-(carboxyethyl)lysine; MOLD = Methylglyoxal-derived lysine dimer; GOLD = Glyoxal-derived lysine. (**B**) Glucose or dicarbonyl compounds can also react with DNA leading to genotoxic adducts such as CEdG and cMGO-dG. CEdG = N^2^-(1-carboxyethyl)-2′-deoxyguanosine; cMGO-dG = 1,N^2^-(1,2-dihydroxy-2-methyl) ethano-dG.

In addition to ROS, the advanced glycation end-products (AGEs)—traditionally studied for their structural and functional impact on proteins—also act as key mediators of DNA damage under hyperglycemic conditions. AGEs arise primarily from the non-enzymatic glycation of proteins, lipids, and nucleic acids by reducing sugars [40]. While protein glycation impairs biological functions such as enzyme kinetics and receptor-mediated signaling, DNA glycation generates genotoxic nucleotide adducts that exacerbate genomic instability [41]. AGEs are primarily generated via the Maillard reaction, in which a reducing sugar, such as glucose, reacts non-enzymatically with amino groups to form Schiff bases. These intermediates rearrange into Amadori products, which further progress to irreversible covalent adducts, giving rise to AGEs (Figure 2). Alternatively, AGEs can be generated through pathways involving highly reactive dicarbonyl compounds, such as methylglyoxal (MGO), glyoxal (GO), glycolaldehyde (GLA), and 3-deoxyglucosone (3-DG). These dicarbonyl compounds are, in turn, produced through the Wolff pathway via metal-ion-catalyzed glucose autoxidation, or alternatively through the degradation of Schiff bases and Amadori products via the Namiki and Hodge pathways, respectively [42,43,44] (Figure 2).

#### AGE-Induced DNA Damage

**Figure 3:**
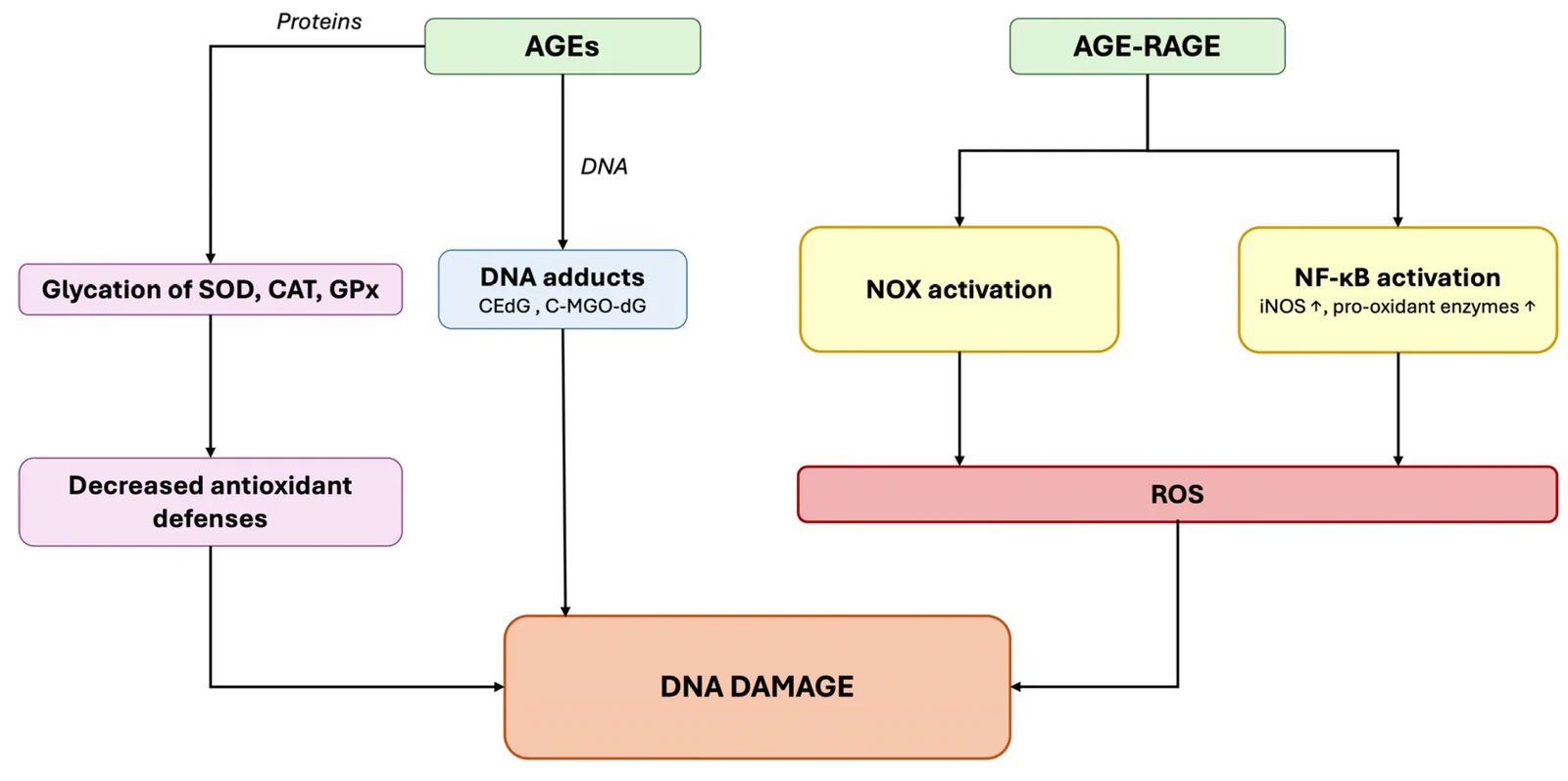
Mechanisms through which AGEs generate DNA damage. Advanced Glycation End-products (AGEs) induce DNA damage through direct and indirect mechanisms. Directly, AGEs form DNA adducts (CEdG and cMGO-dG) and glycate antioxidant enzymes such as superoxide dismutase (SOD), catalase (CAT), and glutathione peroxidase (GPx), impairing cellular defenses. Indirectly, the engagement of the AGE-RAGE axis activates NADPH oxidase (NOX) and NF-κB-driven cytokine production, increasing intracellular ROS levels. Together, structural adducts, decreased antioxidant capacity, and increased ROS converge to induce DNA damage. CEdG = N2-(1-carboxyethyl)-2′-deoxyguanosine; cMGO-dG = 1,N2-(1,2-dihydroxy-2-methyl) ethano-dG. Upward arrows (↑) indicate an increase.

The genotoxic potential of AGEs is primarily driven by two mechanisms: covalent DNA modification and receptor-mediated signaling (Figure 3). The first mechanism involves DNA glycation, which yields genotoxic adducts most notably N^2^-(1-carboxyethyl)-2′-deoxyguanosine (CEdG) and a cyclic diol adduct arising from the direct addition of MGO at the 1, N^2^ position of guanine (cMGO-dG) [45] (Figure 2B). Under physiological conditions, these adducts are routinely processed and repaired by the NER pathway [46]. Conversely, persistent CEdG adducts confer significant mutagenic potential. These adducts induce G:C to T:A and G:C to C:G transversions, promote DNA single-strand breaks (SSBs), and facilitate transposon mobilization [47,48,49]. Furthermore, CEdG accumulation alters local nucleosome organization and compromises chromatin compaction [50]. Additionally, it strongly inhibits transcription in vitro and in mammalian cells [51]. Recent research indicates that MGO adducts on RNA are also clinically relevant, introducing epitranscriptomic modifications that destabilize RNA and impair protein translation [52].

The second mechanism operates through RAGE (Receptor for Advanced Glycation End-products), the primary transmembrane receptor responsible for transducing AGE-mediated pathogenic signals [53]. AGE–RAGE interaction activates the NOX complex, promoting electron transfer from NADPH to molecular oxygen. Electrons released from the nicotinamide ring of NADPH as a hydride ion (H^−^) are transferred by the NOX complex to oxygen, producing superoxide radicals (O_2_^−^•) and subsequently other ROS such as hydroxyl radicals (•OH) [54]. Additionally, RAGE engagement triggers an intracellular signaling cascade that drives the nuclear translocation of the transcription factor NF-κB. Upon activation, NF-κB induces the transcriptional upregulation of pro-oxidant enzymes including NOX subunits and inducible nitric oxide synthase (iNOS), fueling a sustained elevation of intracellular ROS [55].

Direct functional evidence confirms that this receptor-mediated signaling axis actively drives genotoxicity. In porcine kidney models, exposure to AGE-BSA, carboxymethyl-lysine-BSA (CML-BSA), or methylglyoxal-BSA (MGO-BSA) induces marked DNA damage. Crucially, this genotoxic response is completely abolished by either proteolytic cleavage or pharmacological blockade of the extracellular RAGE-binding domain [56]. Furthermore, in human liver and colon cell models, antioxidant treatment or selective inhibition of NF-κB nuclear translocation prevents AGE-induced genomic damage, confirming the central role of this signaling axis [55].

Finally, the non-enzymatic glycation of primary antioxidant enzymes directly impairs their catalytic function, diminishing intracellular scavenging capacity and further increasing overall genomic instability [57].

### 2.3. DNA Repair Impairment

Beyond directly driving DNA damage, hyperglycemia impairs DNA repair mechanisms, creating a permissive environment for persistent genomic instability. Early functional studies demonstrated that peripheral blood lymphocytes from T2D patients exhibited elevated baseline levels of oxidized bases and DSBs. Furthermore, upon exposure to exogenous mutagens such as hydrogen peroxide or doxorubicin, these cells showed greater damage susceptibility and markedly delayed repair kinetics compared to non-diabetic controls, underscoring a compromised baseline repair capacity [58].

Using cultured cell lines, Ciminera et al. [48] identified a specific mechanism through which high glucose levels compromise the NER pathway, the principal system responsible for removing bulky DNA adducts such as CEdG. The authors proposed a model wherein hyperglycemia enhances the activity of 2-oxoglutarate-dependent prolyl hydroxylases (PHDs), promoting degradation of hypoxia-inducible factor-1α (HIF-1α). Because HIF-1α drives the transcription of key NER components, its suppression downregulates NER capacity. This repair deficit leads to persistent unresolved adducts that subsequently collapse into DSBs, triggering downstream activation of the ATM/ATR signaling cascade and γ-H2AX phosphorylation. Accordingly, pharmacological inhibition of PHDs restores HIF-1α -mediated NER transcription and facilitates CEdG clearance [48].

Conversely, hyperglycemia can also overactivate the DDR, which, rather than conferring protection, correlates with persistent damage. Transcriptomic profiling of normal mammary epithelial and breast cancer cells under high-glucose conditions revealed widespread upregulation of major DDR components, including *FANCD2*, key HR genes (*BARD1*, *BRCA2*, *RAD51*, *DNA2*), the mismatch repair gene *MSH6*, and *PARP-1*. Despite the upregulation of these genes, the accumulation of DNA lesions, including DSBs, SSBs, crosslinks, and oxidative lesions, overwhelms the repair machinery. Therefore, the hyperactivated DDR fails to resolve the lesion burden, leading to chronic genomic stress [59].

### 2.4. Antioxidant System Impairment

Diabetes is characterized by a widespread depletion of cellular antioxidant defenses. Under physiological conditions, enzymatic systems—including superoxide dismutase (SOD), catalase (CAT), and glutathione peroxidase (GPx)—cooperate with non-enzymatic antioxidants such as vitamins A, C, E, and B6, reduced glutathione (GSH), and α-lipoic acid to neutralize ROS and prevent oxidative damage [60].

Epidemiological and clinical studies consistently report reduced circulating levels or diminished catalytic activity of SOD, CAT, and GPx in patients with both T1D and T2D [61,62,63,64,65]. This depletion can be due to non-enzymatic glycation of these antioxidant enzymes, which compromises their tertiary structure and catalytic efficiency [66,67] (Figure 3), to ROS-mediated oxidative damage and inactivation of their active sites [68], or to NADPH consumption in the polyol pathway, which limits GSH regeneration and deprives GPx of its essential cofactor [21].

In contrast, some studies report unchanged or elevated levels of these enzymes in both T1D and T2D. However, these discrepancies could be largely attributable to differences in disease duration, glycemic control, and patient comorbidities [69,70,71]; nevertheless, the overall evidence points to a marked decline in antioxidant enzymatic protection.

Non-enzymatic defenses undergo a parallel depletion within the diabetic milieu. Sustained oxidative stress progressively diminishes plasma and tissue levels of key antioxidants, including vitamins C, E, and B6 [65,72,73,74]. However, intervention studies attempting to counteract this deficit through exogenous vitamin supplementation have yielded inconsistent results [75,76,77].

#### Vitamin B6 and DNA Damage in Diabetes

Although not conventionally categorized as a classical antioxidant, growing evidence highlights the important role of vitamin B6 in counteracting oxidative stress. While primary dietary deficiencies are rare, clinical interest in this micronutrient has intensified due to the frequent occurrence of secondary deficiency states induced by common pharmacological agents and associated with diverse widespread pathologies [78].

Vitamin B6 comprises six interconvertible vitamers, pyridoxine (PN), pyridoxal (PL), pyridoxamine (PM), and their respective 5′-phosphate derivatives (PNP, PLP, and PMP). Its biologically active form, pyridoxal 5′-phosphate (PLP), serves as an essential coenzyme in approximately 150 enzymatic reactions. Beyond its metabolic functions, PLP exerts a multifaceted protective role in genomic integrity [78]. Within one-carbon metabolism, PLP functions as a cofactor in pathways essential for both nucleotide biosynthesis and reduced glutathione (GSH) synthesis. Furthermore, vitamin B6 can directly quench ROS and inhibit AGE formation [79,80].

Decreased circulating vitamin B6 levels are routinely observed in patients with T1D or T2D, as well as in those with micro- and macrovascular complications [74,81]. Studies in *Drosophila* contributed to clarifying the role of vitamin B6 in T2D. PLP depletion, induced either by mutations in genes involved in its biosynthesis—such as *pyridoxal kinase* (*Pdxk*) and *pyridoxine/pyridoxamine oxidase* (*Sgll^PNPO^*)—or by pharmacological antagonists, leads to hyperglycemia and insulin resistance, increased ROS levels, chromosome aberrations (CABs), DSBs, and accelerated AGE accumulation [2,82,83]. Notably, these findings established that vitamin B6 deficiency is not merely a secondary consequence of metabolic dysfunction but can actively drive diabetic pathogenesis.

Consistent with the ability of vitamin B6 to interfere with multiple stages of glycation [43], low PLP levels fail to mitigate ongoing AGE accumulation, thereby exacerbating oxidative stress and genomic instability in *Drosophila* and human cells [82]. Crucially, exogenous supplementation with PLP or α-lipoic acid rescues DNA damage and normalizes AGE levels across distinct *Drosophila* models of T2D [84,85]. Furthermore, transgenic expression of human *PDXK* in flies lacking the endogenous ortholog fully rescues CABs and prevents AGE formation, confirming the evolutionary conservation of this protective mechanism [82].

## 3. Evidence of DNA Damage in Diabetic Cells

Experimental studies utilizing cellular and animal models have clearly established a direct genotoxic potential of hyperglycemia. Exposure to high glucose concentrations (30 mmol/L) has been shown to induce DNA strand breaks in cultured human endothelial cells [86]. Similar results were obtained using mouse and human renal cells [87]. Furthermore, exposing normal cultured human cells to such high glucose concentrations increased mutagenesis at specific loci, such as *hypoxanthine–guanine phosphoribosyltransferase* (*HPRT*) and *thymidine kinase* (*TK*) [88]. In line with these results, Lee et al. [89] found that high glucose concentrations in mouse embryos increased mutagenesis both in vitro and in vivo.

At the molecular level, exposure of endothelial cells to high glucose stimulated ROS overproduction through PKC-dependent activation of NOX and resulted in the accumulation of 8-oxodG [90]. Consistently, elevated levels of 8-OHdG were found in mononuclear cells of T1D and T2D patients [91].

Moreover, increased serum and urinary levels of 8-oxodG, which significantly correlated with poor glycemic control, have been reported in both forms of diabetes [92,93,94,95].

Individuals with diabetes also display increased plasmatic levels of MGO [96] and consistently, CEdG levels are significantly increased in animal models as well as in patients with T1D and T2D [41,45,97,98].

Case–control studies utilizing the comet assay reveal a high frequency of DNA strand breaks—especially in T2D individuals with poor glycemic control—correlated with inflammatory and oxidative stress biomarkers such as NOX, IL-6, and urinary albumin [99]. Furthermore, because unrepaired DSBs can drive chromosome damage [100], research demonstrates elevated levels of stable chromosomal aberrations in the peripheral lymphocytes of T2D patients [101,102].

Additionally, T1D and T2D patients exhibit an elevated frequency of sister chromatid exchange (SCE) and micronuclei compared to healthy controls [103,104]; SCEs occur during DNA replication, when two sister chromatids break and rejoin. Although they do not typically produce dangerous cellular lesions, they serve as extremely sensitive indicators of DNA replication stress and mutagenic exposure. Similarly, micronuclei form during cell division as a result of DNA damage or segregation errors, acting as reliable biomarkers of genomic instability.

Chromosome aberrations, DNA damage, ROS and AGE accumulation have also been observed in *Drosophila* T2D models [82,83,84,85,105].

Diabetes can also affect telomere length [106]. Telomere shortening has been correlated with both T1D and T2D across several studies [107]. Because telomere sequences are rich in guanine residues—which are particularly vulnerable to oxidative stress—uncontrolled diabetes accelerates this damage. When telomeres reach a critically short length, they may induce senescence and apoptosis, potentially accelerating cell death and contributing to diabetic complications; however, the exact extent of this impact remains to be fully established [108].

Further confirming the link between diabetes and oxidative stress, a recent high-throughput study demonstrated that patients with T1D and T2D exhibit widespread transcriptional alterations in the levels of mRNAs and microRNAs associated with oxidative stress response, DNA repair, the antioxidant system, endoplasmic reticulum stress, and mitochondrial dysfunction [109].

## 4. Consequences of DNA Damage in Diabetes

Over time, unresolved genomic alterations contribute to the development of micro- and macrovascular diabetic complications by triggering complex pathogenic cascades. Concurrently, persistent genomic instability directly favors malignant transformation. Moreover, DNA damage can contribute to male infertility associated with diabetes. Advancements in diabetes care have extended life expectancy, uncovering a range of other emerging complications—from cognitive decline to affective disorders, obstructive sleep apnea, and liver disease [110]. More data on these complications are expected to emerge in the future, yet their analysis remains outside of the scope of this review.

### 4.1. Diabetes Complications

#### 4.1.1. Role of Oxidative Stress in Diabetes Complications

Microvascular damages include retinopathy, nephropathy, and peripheral neuropathy, which can cause vision loss (up to blindness), chronic kidney disease (requiring dialysis or transplantation), and nerve damage with a risk of ulcers and amputations. On the other hand, macrovascular complications can result in coronary heart disease, stroke, and peripheral vascular disease [9].

The progressive accumulation of oxidative DNA damage markers such as 8-oxodG and DSBs strongly correlates with diabetes progression. For example, urinary excretion of 8-oxodG and its content in mononuclear cells are higher in T2D patients with complications compared to those without complications and correlate with the glycated hemoglobin value [92,93]. Similar results were found by assessing plasmatic levels of 8-oxodG [111,112]. Moreover, in T2D patients affected by diabetic nephropathy, DNA fragmentation damage correlates directly with microalbuminuria levels [113]. Consistently, elevated serum and urinary 8-oxodG concentrations have been associated with rapid glomerular filtration rate decline in T2D [114]. Oxidative damage is also evident in other diabetic complications. 8-oxodG levels are significantly higher in T2D patients with proliferative diabetic retinopathy than in those with the non-proliferative stage [115]. Furthermore, overall oxidative DNA damage correlates closely with the severity of diabetic polyneuropathy in T2D [116]. Finally, elevated 8-oxodG levels in T2D patients affected by cardiovascular diseases have been associated with an increased mortality risk [117].

**Figure 4:**
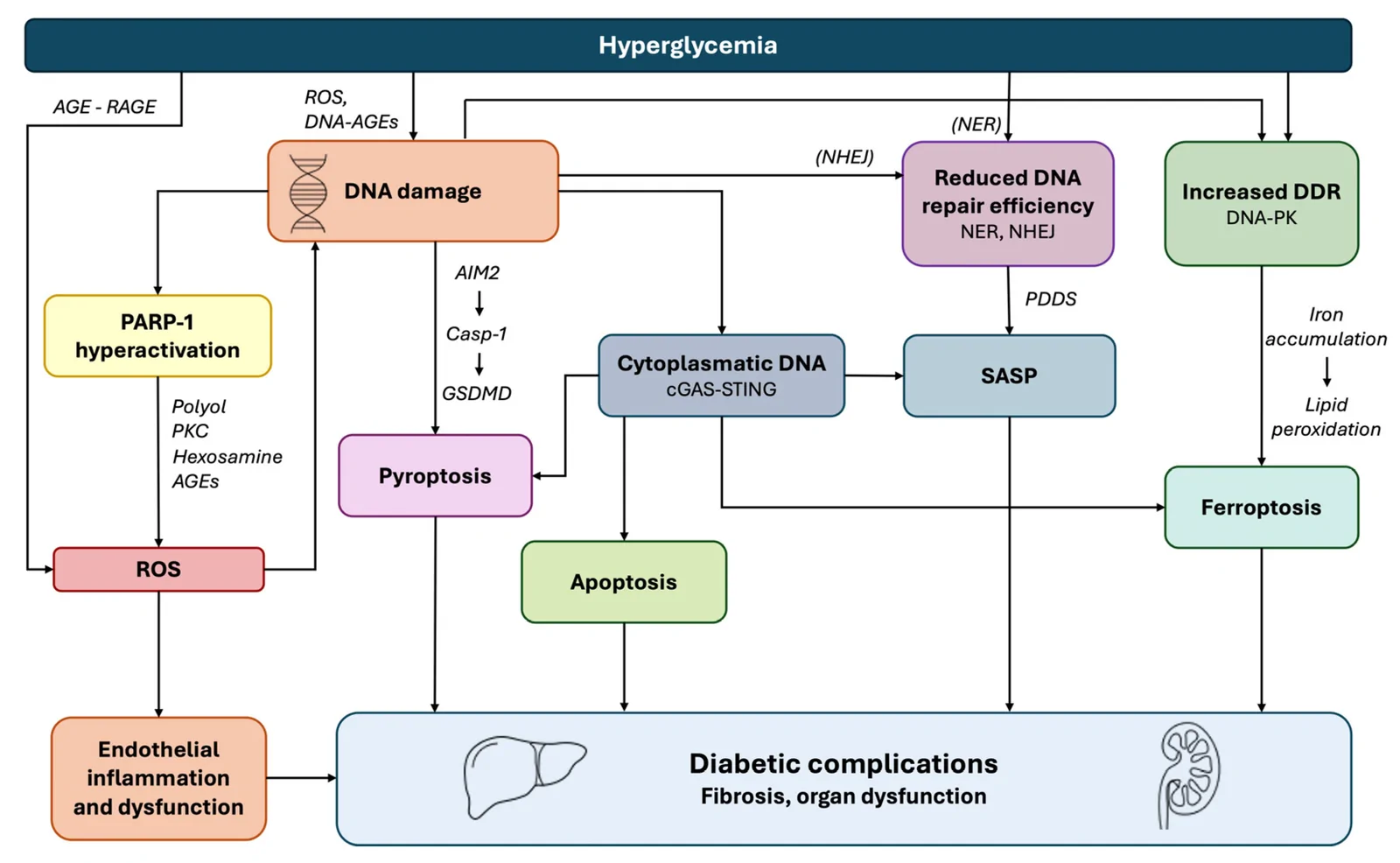
Pathways linking hyperglycemia-induced DNA damage, altered DNA repair, and cell fate to diabetic complications. Hyperglycemia drives systemic organ dysfunction through distinct pathways triggered by DNA damage. Primarily, DNA damage—promoted by ROS and DNA-AGE adducts—activates PARP-1, triggering secondary pathways (polyol, PKC, hexosamine, and AGEs) that generate further ROS that induce endothelial inflammation and dysfunction and simultaneously exacerbate DNA damage. Independently, AGE-RAGE signaling contributes to ROS generation and downstream endothelial inflammation and DNA damage. Unrepaired DNA damage directly triggers pyroptosis via the AIM2–caspase-1–GSDMD cascade. Reduced DNA repair efficiency, particularly through non-homologous end joining (NHEJ), results in persistent DNA damage signaling (PDDS) that promotes the senescence-associated secretory phenotype (SASP). Hyperglycemia also enhances DNA damage response signaling through DNA-PK, driving iron accumulation and lipid peroxidation to induce ferroptosis. Cytoplasmic DNA accumulation activates cGAS-STING signaling, which independently converges to drive apoptosis, pyroptosis, SASP, and ferroptosis. Together, these pathogenic pathways mediate tissue fibrosis and organ failure in diabetic complications.

Regarding the mechanisms linking DNA damage to diabetes complications [16,118], Brownlee and collaborators proposed a model wherein mitochondrial-derived ROS act as the primary triggers of DNA damage (Figure 4). In particular, DNA strand breaks induced by superoxide cause the hyperactivation of PARP-1 which drives further ROS generation (as described in Section 2.1.1) and triggers tissue and vascular damage [119,120].

Accordingly, diabetic models of rats and mice exhibited elevated PARP-1 transcript levels in both the renal cortex and the retina [121,122]. Consistent with the pathogenic role of PARP-1 activation, pharmacological inhibition of PARP-1 attenuates diabetic nephropathy in leptin receptor-deficient (*db*/*db*) mice [121] and retinopathy in diabetic rats [122], restores motor and sensory nerve conduction velocity in neuropathy in streptozotocin-induced diabetic rats [123], and reduces atherosclerotic calcification in diabetic *ApoE^−/−^* mice [124].

#### 4.1.2. Role of AGEs in Diabetes Complications

Several attempts to mitigate complications with antioxidant supplementation have yielded inconsistent results, prompting research into alternative drivers of diabetic secondary diseases [125]. Among these, AGEs have been pinpointed as major contributors to diabetic complications, mainly through the production of non-mitochondrial ROS and direct glycation of proteins, lipids, and DNA (Figure 4). Consistent with these findings, AGEs accumulate extensively in serum, vascular walls, the retina, and renal tissues. Accordingly, animal studies demonstrate that exposure to high levels of exogenous AGEs promotes renal and vascular complications [126,127].

Glycation of proteins alters their structure and function and produces cross-links between components of the extracellular matrix basement membrane, thus permanently destroying cellular structure. In contrast, DNA glycation yields adducts that produce mutations or stall replication forks, leading to secondary DSBs that ultimately trigger vascular cell apoptosis and persistent tissue damage [98]. Consistently, MGO adducts have been proposed as prognostic biomarkers for diabetic kidney disease in T1D patients [128]. T2D complications are also associated with MGO adducts on RNA, which reduce mRNA stability and impair protein translation [52].

The interaction between AGEs and their cellular receptors plays an important role in the pathogenesis of diabetic complications. It has been extensively demonstrated in animal models that AGE–RAGE interaction can induce downstream signaling pathways leading to increased ROS levels and consequent endothelial cell injury and apoptosis [129]. ROS can also attack DNA, increasing genome instability [98].

In diabetic retinopathy, the AGE-RAGE interaction accelerates pericyte apoptosis; in nephropathy, it leads to tubulointerstitial ischemia; and in neuropathy, it promotes cellular apoptosis and nerve fiber loss. Administration of antioxidants in diabetic mice suppresses ROS and prevents caspase-3-mediated retinal neuronal apoptosis [130], while RAGE knockout in diabetic ApoE-deficient mice significantly reduces atherosclerotic plaque area, downregulates NF-κB signaling, and attenuates NOX expression [131].

#### 4.1.3. DNA Damage-Driven Pyroptosis in Diabetic Organ Dysfunction

Recent studies revealed that DNA damage can trigger novel signaling pathways that significantly contribute to organ dysfunction in diabetic complications (Figure 4). Driven by these pathways, pyroptosis, a form of lytic, pro-inflammatory cell death, plays a central role [132]. The canonical pyroptotic cascade relies on the assembly of an inflammasome complex comprising a sensor receptor (such as NLR family proteins, AIM2, or Pyrin), the ASC adapter protein, and pro-caspase-1. This assembly drives the autoproteolytic activation of caspase-1, which subsequently cleaves Gasdermin D (GSDMD). The resulting N-terminal fragment of GSDMD oligomerizes to form plasma membrane pores, ultimately leading to osmotic lysis and the release of pro-inflammatory cytokines [133]. Intriguingly, emerging evidence in diabetic nephropathy demonstrates that nuclear DNA damage can act as an upstream trigger for this cascade. Upon nuclear localization and recognition of damaged DNA, AIM2 engages the inflammasome complex to drive downstream pore execution and tissue injury. Consistently, AIM2 upregulation in the renal cortex of *db*/*db* mice as well as in high glucose-induced Human Kidney-2 cells (HG-HK-2) correlates directly with the extent of DNA damage, whereas ROS scavenging or the knockdown of AIM2 or GSDMD effectively prevents pyroptotic cell death and attenuates renal injury in HG-induced HK-2 cells [134].

#### 4.1.4. Role of DNA Repair in Diabetes Complications

A new perspective places greater emphasis on the role of DNA repair in complications, thereby favoring the long-term persistence of damage and impacting vascular pathology in T1D and T2D [10] (Figure 4). Early studies suggested that T1D individuals suffering from impaired DNA repair exhibit an increased susceptibility to disease-related complications [135]. In diabetic nephropathy, severe 8-oxodG accumulation coincides with a marked downregulation of human 8-oxoguanine glycosylase 1 (hOGG1), a pivotal enzyme in base excision repair, confirming that compromised repair capacity accelerates tissue injury. The translational relevance of this protective mechanism is highlighted by clinical transcriptomic profiling in long-standing T1D patients, revealing a significant enrichment of DNA repair pathways exclusively in patients not progressing through vascular complications (non-progressors) compared to those developing progressive damage (progressors) [136].

#### 4.1.5. Impaired DNA Repair and Fibrosis

When persistent DNA damage overwhelms repair machinery, it triggers downstream signaling pathways that shift cell fate toward chronic dysfunction or programmed death (Figure 4). A prolonged state of unrepaired DNA damage, particularly the accumulation of DSBs, impairs the NHEJ repair system and induces persistent DNA damage signaling (PDDS) in T1D and T2D mouse models [10,137]. PDDS drives parenchymal and vascular cells into premature senescence, causing them to adopt a senescence-associated secretory phenotype (SASP) characterized by the release of pro-inflammatory cytokines, chemokines, and matrix-degrading enzymes that promote local tissue remodeling and telomere dysfunction. In the kidney, SASP accumulation directly promotes interstitial fibrosis and progressive functional loss, whereas in the cardiovascular system, SASP impairs the regenerative potential of cardiomyocytes, aggravating macrovascular dysfunction. Targeting these senescent cells with senolytic therapies is an emerging approach to arrest or reverse fibrotic organ decline [10].

#### 4.1.6. Impairment of DDR Promotes Ferroptosis

On the other hand, severe genomic damage can hyperactivate the DDR, initiating downstream signaling pathways that induce ferroptosis [138] (Figure 4). This iron-dependent, non-apoptotic form of regulated cell death is driven by the lethal accumulation of membrane lipid peroxides and has recently emerged as a relevant contributor to organ dysfunction in diabetic complications. In T2D cardiomyopathy, hyperglycemia induces an aberrant DDR that activates the DNA-dependent protein kinase (DNA-PK) complex, comprising PRKDC, XRCC5, and XRCC6. This cascade exacerbates membrane lipid peroxidation, triggering endothelial ferroptosis, thereby compromising microvascular permeability and impairing cardiac function. Pharmacological inhibition of DNA-PK suppresses lipid peroxidation and rescues cardiac function [139].

#### 4.1.7. Role of cGAS-STING Pathway in Diabetes Complications

In recent years, the role of the cyclic GMP-AMP synthase–STimulator of Interferon Genes (cGAS-STING) pathway in diabetic complications has increasingly emerged [140,141]. This pathway is activated when nuclear or mitochondrial DNA fragments are released into the cytosol under severe metabolic and oxidative stress conditions. Aberrant activation of the cGAS-STING axis contributes to inflammation and organ dysfunction by promoting cell death programs and senescence-associated responses such as apoptosis, ferroptosis, pyroptosis and fibrosis [140,141,142] (Figure 4). Consistently, in retinopathy drug-mediated suppression of STING decreases cytosolic DSB accumulation and mitochondrial injury in vitro, while pharmacological blockade successfully improves retinal pigment epithelium (RPE) dysfunction and vascular alterations in vivo [143].

#### 4.1.8. Metabolic Memory

The long-term persistence of diabetic complications, even after achieving tight glycemic control, is attributed to the phenomenon of metabolic memory. Hyperglycemia, oxidative stress, and transient genotoxic events induce epigenetic alterations, including DNA methylation changes, post-translational histone modifications, and non-coding RNA dysregulation. These persistent epigenetic marks perpetuate vascular endothelial dysfunction, sustained NF-κB activation, and chronic pro-inflammatory signaling, explaining why early metabolic insult exerts pathological effects long after normoglycemia is restored [144]. Although further clinical and mechanistic studies are required to elucidate the molecular basis of metabolic memory and its contribution to diabetes progression, targeting epigenetic mechanisms through behavioral interventions, nutritional strategies, and pharmacological approaches represents a compelling frontier for future research [145].

### 4.2. Cancer and Diabetes

Epidemiological evidence consistently demonstrates that diabetes is an independent risk factor for various malignancies [146,147]. Moreover, in patients with diabetes, cancer frequently exhibits more aggressive clinicopathological features and is associated with reduced overall survival [148]. Although methodological challenges—such as detection bias, reverse causality, and confounding factors—can complicate the research, long-term data confirm a clinically significant association that demands targeted public health interventions [110].

An umbrella review of observational and Mendelian randomization studies exploring the association between T2D and cancer indicated that the most robust evidence was found for colorectal cancer, with strong associations also documented for pancreatic, endometrial, breast, hepatic, and urinary bladder cancers [147]. In contrast, although an association has been proposed between T2D and a reduced incidence of prostate cancer, current findings remain inconsistent, leaving the existence of a causal link uncertain [149].

For T1D, fewer data are available; however, a meta-analysis of observational studies revealed a modest overall increase in cancer risk particularly for thyroid, stomach, lung, pancreatic, liver, ovarian, and kidney cancers [150].

The precise molecular mechanisms linking diabetes to tumor initiation remain to be fully elucidated; proposed drivers include the mitogenic and anti-apoptotic actions of insulin and insulin-like growth factor 1 (IGF-1), steroid hormone dysregulation, chronic low-grade inflammation, and hyperglycemia [151].

Since Warburg first identified the link between high glucose uptake and tumorigenesis [152], extensive evidence has confirmed that hyperglycemia directly accelerates tumor development. Specifically, mouse models have demonstrated that hyperglycemia can stimulate tumor growth and metastasis simultaneously or even promote metastatic dissemination independently [153,154,155].

ROS induced by hyperglycemia modulate multiple signaling pathways driving cell proliferation, migration, invasion, and resistance to apoptosis, in addition to reshaping the tumor microenvironment to facilitate dissemination in mouse models of diabetes [156,157].

**Figure 5:**
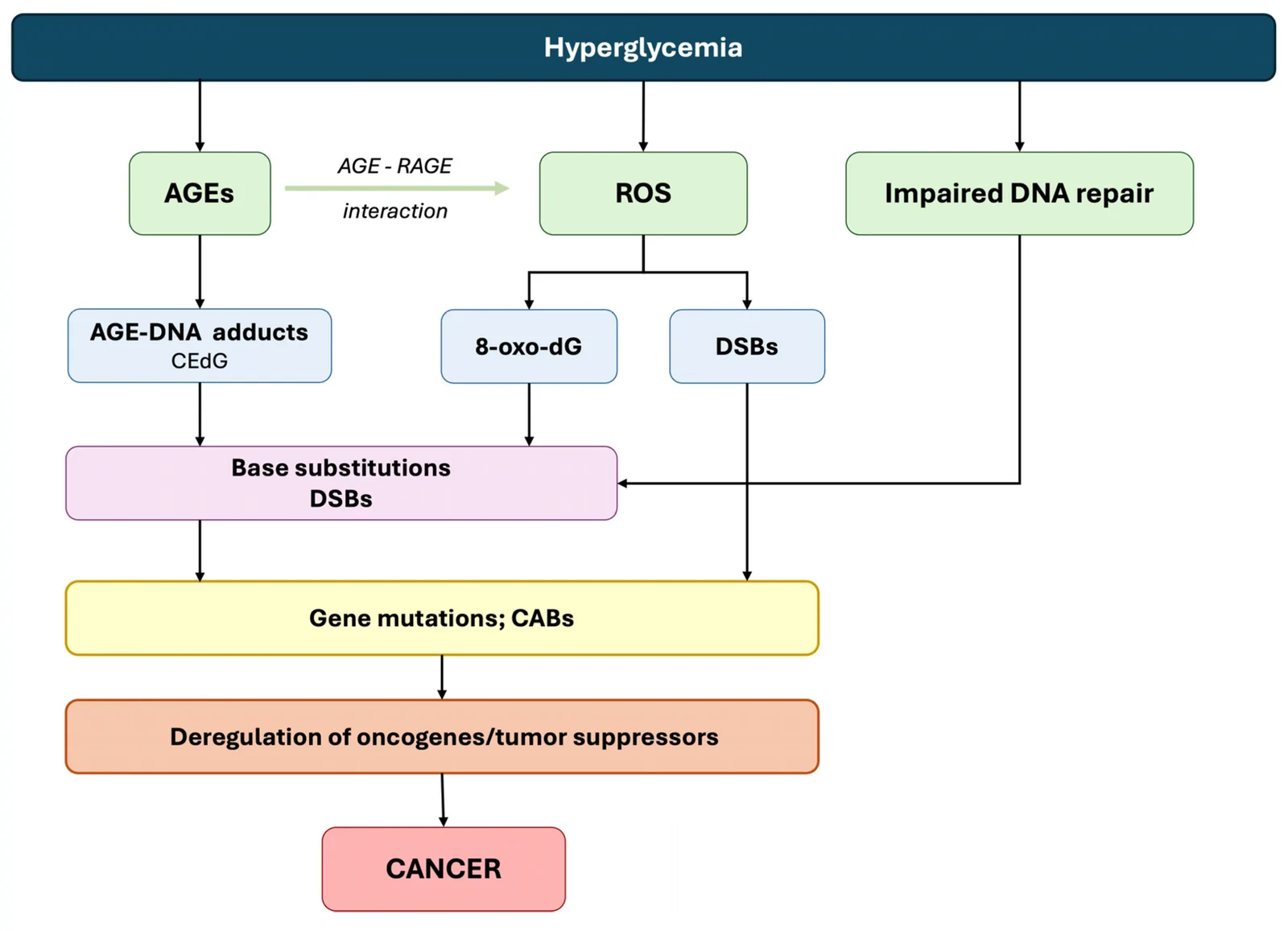
Mechanisms linking hyperglycemia to genomic instability and carcinogenesis. Hyperglycemia promotes oncogenesis through three distinct pathways. First, accumulation of AGEs leads to AGE-DNA adduct formation (CEdG) and stimulates ROS production via AGE-RAGE interaction. Second, direct ROS accumulation induces oxidatively damaged bases (8-oxo-7,8-dihydro-2′-deoxyguanosine, 8-oxo-dG) and double-strand breaks (DSBs). Third, hyperglycemia impairs DNA repair mechanisms, exacerbating base substitutions and DSBs. Accumulated adducts, base substitutions, and DSBs lead to gene mutations and chromosome aberrations (CABs), which alter oncogene and tumor suppressor expression to drive cancer development.

Given the well-established link between genomic instability and cancer—first hypothesized by Boveri and later validated through the identification of tumor-associated chromosomal abnormalities [158]—recent research has increasingly focused on hyperglycemia-induced DNA damage as a key driver of heightened cancer risk in diabetic patients. As previously mentioned, oxidative stress can produce lesions ranging from oxidized bases to DSBs, thus potentially producing gene mutations, stalling replication forks, and leading to chromosomal aberrations such as deletions, duplications, translocations, and inversions. These chromosomal aberrations can dysregulate key oncogenes and tumor suppressors, triggering cancer onset and development (Figure 5).

Additionally, persistent hyperglycemia leads to the non-enzymatic glycation of proteins, lipids and DNA, driving the progressive accumulation of AGEs. Thus, the genotoxic potential of AGEs is increasingly recognized as a key mechanistic link between diabetes and cancer (Figure 5). AGEs promote genomic instability directly by forming adducts with DNA, and indirectly by engaging their receptor. RAGE activation triggers chronic inflammation and further amplifies downstream ROS production. The presence of AGEs has been demonstrated in several human tumors and a positive correlation between RAGE expression and disease progression has been shown across diverse cancers including breast, gastric, ovarian, pancreatic malignancies and melanoma [159,160,161,162,163,164]. In breast cancer cells, high RAGE expression strongly correlates with poor prognosis [165], whereas in human pancreatic cancer specimens, RAGE is markedly overexpressed compared to adjacent normal ducts [163]. Demonstrating a functional in vivo link, exogenous administration of the specific AGE species $N^{\epsilon }$-(carboxymethyl)lysine (CML) to pancreatic cancer-prone mice upregulated RAGE within pancreatic intraepithelial neoplasias, prompting their rapid progression to invasive cancer [159].

In addition to DNA damage, increasing importance is attributed to DNA repair impairment as a cancer driver in diabetes (Figure 5). For example, in renal tubular cells from diabetic patients with renal cell carcinoma, hyperglycemia hyperactivates the Akt/mTOR pathway, which downregulates the DNA repair enzyme 8-oxoguanine DNA glycosylase 1 (OGG1). Because OGG1 is required for excision-repair of 8-oxodG, its suppression under chronic hyperglycemic conditions causes persistent oxidative DNA damage that acts as a tumor initiator [166].

Accordingly, long-term exposure of hepatocytes to high glucose concentrations reduced mRNA expression of BER genes, leading to accumulated DNA damage [167]. Furthermore, the clinical consequences of this altered repair capacity extend to treatment outcomes; specifically, increased chemo- and radio-resistance in renal cell carcinoma following high glucose exposure has been attributed to this compromised repair phenotype [168].

Beyond BER, hyperglycemia impacts other repair pathways. Ciminera et al. [48] found that in human cells and mouse models hyperglycemia impairs the NER system by preventing the removal of CEdG adducts, proposing that the resulting genomic instability increases cancer risk and enhances susceptibility to endogenous and exogenous DNA-damaging agents.

In line with these observations, a recent metabolomic and transcriptomic analysis across breast cancer animal models, patient tissues, and cell cultures revealed a proinflammatory metabolome and a signature of DNA repair deficiency triggered by hyperglycemia, alongside high levels of DNA lesions. These findings led the authors to speculate that diabetes-induced cancer cells might be more vulnerable—and consequently have a better therapeutic response—to DNA repair inhibitors [154].

Conversely, while repair downregulation is a prominent driver, increased DDR activity has also been proposed as a mechanism that promotes cancer by managing high levels of genome instability [59]. Reflecting this alternative pathway, studies have observed a significant upregulation of 36 DDR genes in ductal and lobular breast carcinomas in comparison to normal breast cells [169].

Reduced antioxidant defenses can also trigger genome instability in diabetes, thus increasing the cancer risk. Utilizing *Drosophila melanogaster* as a model organism, recent studies have demonstrated that PLP depletion causes DNA damage exacerbated by glucose, fructose and sucrose [82]. Additionally, depleting PLP in various T2D *Drosophila* models severely exacerbates genomic instability. Specifically, diabetic cells treated with the vitamin B6 antagonist 4-deoxypyridoxine (4-DP) exhibited a roughly threefold increase in structural chromosome aberrations compared to non-diabetic controls [105]. These findings highlight a marked vulnerability of diabetic cells to the loss of vitamin B6—a crucial antioxidant and enzymatic cofactor. Furthermore, these results suggest that reduced vitamin B6 levels in diabetes may contribute to elevated cancer risk in diabetic patients.

Dissecting the complex molecular interplay between diabetes and cancer is crucial to fully understanding how diabetic metabolic dysfunction shapes the mutational landscape of tumor initiation and progression.

### 4.3. Male Infertility and Diabetes

Both T1D and T2D are strongly associated with male infertility. Oxidative stress represents an important pathogenic mechanism, with epigenetic modifications also playing a role [14]. Spermatozoa are particularly vulnerable to oxidative damage due to their minimal cytoplasmic volume—which severely restricts intracellular antioxidant defenses—and a plasma membrane exceptionally enriched in polyunsaturated fatty acids (PUFAs). While physiological levels of ROS are required for crucial sperm functions, including capacitation and the acrosome reaction, the diabetic metabolic state disrupts this homeostatic balance [170]. Pathologically elevated ROS levels impair sperm functionality through multiple converging pathways, such as lipid peroxidation, loss of membrane fluidity, mitochondrial dysfunction, and impairment of the hypothalamic–pituitary–gonadal (HPG) axis, which suppresses intratesticular testosterone synthesis [171].

A major clinical consequence of this diabetes-induced oxidative stress is sperm DNA fragmentation (SDF), a critical factor in male subfertility that directly compromises embryonic development, implantation rates, and pregnancy outcomes [172,173,174]. Clinical evidence demonstrates that up to 53% of diabetic men exhibit pathologically elevated SDF, compared to 32% of healthy controls [14]; nevertheless, the precise mechanisms responsible for SDF origin remain incompletely understood.

Cells respond to diabetes-induced damage at distinct stages along the male reproductive tract. During early spermatogenesis within the seminiferous tubules, germ cells retain active checkpoints and functional apoptotic machinery; massive oxidative or metabolic stress at this stage triggers classical apoptosis, resulting in germ cell death, Sertoli cell phagocytosis, and a clinically evident decline in sperm count [175]. As development progresses into late spermiogenesis, chromatin remodeling required for DNA compaction relies on topoisomerase II to generate transient double-strand breaks that relieve torsional strain while protamines replace histones [174]. In the diabetic testes, high oxidative stress and compromised repair mechanisms prevent the ligation of these breaks, yielding immature spermatozoa with poorly condensed, histone-retained chromatin [176]. Upon exiting the testis, mature spermatozoa discard most of their cytoplasm, thereby losing their apoptotic enzymes and DNA repair machinery. Consequently, as these cells migrate through an epididymal microenvironment enriched with ROS and AGEs, they can no longer undergo complete cell death. Instead, oxidative stress and ROS-activated endonucleases trigger incomplete or “abortive” apoptosis, allowing morphologically intact and motile spermatozoa to enter the ejaculate while carrying extensive nuclear strand breaks [174].

Furthermore, ROS-driven lipid peroxidation of the PUFA-rich plasma membrane generates reactive radicals and harmful aldehydes capable of forming covalent DNA adducts, further amplifying genomic instability. The marked elevation of ROS, AGEs, and soluble RAGEs within the seminal plasma, reproductive tract, and spermatozoa of T1D and T2D diabetic males underscores their active involvement in inducing this genomic damage [176,177].

Collectively, these findings demonstrate that diabetes directly drives sperm genomic damage through a combination of impaired protamination, epididymal oxidative stress, and the failure of mature spermatozoa to undergo complete apoptosis. Consequently, SDF evaluation represents an indispensable biomarker in diabetic men, supporting integrated clinical strategies that combine strict glycemic control, targeted antioxidant therapy, and early genomic screening to safeguard male reproductive health.

### 4.4. Translational Approaches and Emerging Therapies to Mitigate DNA Damage

Despite the efficacy of conventional glucose-lowering therapies such as insulin, sodium–glucose cotransporter 2 (SGLT2) inhibitors and glucagon-like peptide-1 (GLP-1) receptor agonists, many patients continue to suffer from progressive micro- and macrovascular diabetic complications due to persistent inflammation, oxidative stress and DNA damage. Consequently, targeting genome instability and pathways leading to oxidative stress represents a promising strategy to mitigate chronic organ injury. Although encouraging results from preclinical studies have laid the groundwork for clinical translation, several challenges must be resolved before proceeding to clinical trials.

Chronic hyperglycemia in diabetes generates ROS and reactive dicarbonyls, causing severe nuclear and mitochondrial DNA damage that accelerates microvascular and macrovascular complications and increases cancer risk and male sterility. Key biomarkers include 8-oxodG for oxidative base damage, γ-H2AX for double-strand DNA breaks, and dicarbonyl-derived AGE-DNA adducts. Functional tools like the comet and micronucleus assays further quantify systemic strand breaks and chromosomal instability. These biomarkers offer valuable targets for early risk assessment and therapeutic monitoring in diabetic nephropathy, retinopathy, and cardiovascular disease.

Pharmacological inhibition of PARP-1 using small molecules such as PJ34 has demonstrated extensive organ-protective effects across diverse diabetic rodent models of T1D and T2D, including streptozotocin-induced diabetic rats, leptin receptor-deficient (*db*/*db*) mice, and *ApoE*^−/−^ mice. It has been shown that in these models, PARP-1 blockade restores vascular responsiveness, improves cardiac performance, and alleviates diabetic nephropathy, retinopathy, neuropathy, and atherosclerotic calcification [27,121,122,123,124]. However, lifelong management using systemic PARP-1 inhibition raises safety concerns in humans due to potential toxicity and off-target effects and interference with physiological DNA repair mechanisms. To mitigate these issues, it has been proposed to use PARP-1 inhibitors in combination with other compounds [122].

Modulation of the RAGE axis represents another widely explored avenue. Preclinical studies in diabetic mouse and rat models have demonstrated that RAGE inhibition, achieved through knockout, neutralizing antibodies, small-molecule inhibitors, or DNA aptamers, can attenuate atherosclerotic plaque formation, suppress NF-κB-mediated inflammatory signaling and NOX-dependent oxidative stress, prevent endothelial-to-mesenchymal transition, and reduce renal fibrosis and podocyte injury [129]. Soluble RAGE (sRAGE) and RAGE antagonist peptides (RAPs) similarly restore vascular and tissue function in rodents. However, human clinical application of sRAGE requires large amounts of recombinant protein, substantially increasing treatment costs [178]. To date, RAGE-targeted strategies have not advanced to approved clinical trials or treatments for diabetic complications, largely due to receptor redundancy and uncertainties regarding long-term safety during chronic administration.

In vitro and in vivo studies demonstrate that NOX enzymes play a pivotal role in diabetic complications, highlighting their potential as therapeutic targets. Preclinical research using the dual NOX1/NOX4 inhibitors GKT137831 and GKT136901 has shown renoprotective effects in T1D and T2D mouse models, respectively, by reducing oxidative stress and attenuating renal injury [179,180,181]. In contrast, a phase II clinical trial evaluating GKT137831 in type 2 diabetes patients with nephropathy failed to reduce albuminuria. However, Urner et al. [179] reported that a trial is ongoing in T1D patients to evaluate whether long-term treatment with GKT137831 can provide renoprotection.

Regarding diabetic retinopathy, GKT137831 decreased hypoxia-driven ROS elevation, pro-inflammatory marker expression, leukocyte infiltration, VEGF-A expression, and retinal vascular leakage in a rat pup model of oxygen-induced retinopathy [182]. However, poor isoform specificity and off-target effects slow clinical progress; therefore, developing more selective inhibitors of NOX isoforms remains the most promising avenue for future therapeutic strategies.

Pharmacological inhibition of PHDs using the specific PHD inhibitor daprodustat (GSK1278863) stabilized HIF-1α and increased NER gene/protein expression and DNA repair efficiency in cultured cells under high-glucose conditions [48].

Other PHD inhibitors, including enarodustat (JTZ-951), roxadustat (FG-4592) and its analog, FG-4497, have also demonstrated beneficial effects on glucose metabolism and diabetes-associated complications in preclinical studies. In obese T2D BTBR *ob*/*ob* mice, enarodustat improved glucose tolerance, reduced adiposity, decreased proteinuria, and preserved renal microvascular integrity [183]. It also counteracted diabetes-associated alterations in renal energy metabolism and reduced oxidative stress in experimental models of early diabetic kidney disease [184]. Similarly, FG-4497 improved glucose and lipid metabolism and reduced adiposity in diet-induced obese mice [185]. Roxadustat increased glycolysis, reduced mitochondrial respiration, and enhanced insulin-stimulated glycogen synthesis in primary human myotubes derived from donors with T2D [186].

Although several PHD inhibitors have been approved or have advanced into late-stage clinical trials for the treatment of anemia associated with chronic kidney disease, their efficacy as therapeutic agents for diabetes has yet to be established in dedicated clinical trials [185].

Recent studies establish that the nuclear isoform of RAGE (nRAGE) directly mediates DNA double-strand break repair via ATM-mediated phosphorylation and MRE11 recruitment [187]. To suppress pathological DNA damage response (DDR) gene overexpression, delivery of a phosphomimetic nRAGE mutant (RAGE-S376E/S389E) has been evaluated in long-term diabetic mice. This intervention diminished γ-H2AX foci, successfully attenuating DNA damage, cellular senescence, the senescence-associated secretory phenotype (SASP), and organ fibrosis [137]. However, no clinical trials have been undertaken to date.

cGAS-STING inhibitors, such as C-176 and H-151, have shown promising anti-inflammatory effects in preclinical models of diabetic complications. Specifically, C-176 reduced NF-κB-driven inflammation and accelerated wound healing [140], while H-151 attenuated myocardial ischemia–reperfusion injury and improved cardiac function in diabetic mice [188]. Although targeting this pathway holds significant therapeutic promise, its clinical translation faces multiple obstacles, including cell- and tissue-specific challenges, potential risks of systemic immunosuppression and infection, functional redundancy with alternative DNA sensors, limited biomarker availability, and drug-delivery barriers. Addressing these issues will require future investigations focused on cell-targeted interventions, multi-target combinations, biomarker panel development, and advanced single-cell and spatial omics to resolve spatiotemporal pathway dynamics [189].

Inhibiting DNA-PK with NU7441 or M9831 molecules in mice with T2D-associatedcardiomyopathy restores cardiac systolic and diastolic parameters, while maintaining microvascular endothelial barrier integrity [139]. However, clinical translation will require careful dose optimization to balance therapeutic benefits against the risk of disrupting baseline DNA repair [190].

The emerging role of the SASP in diabetic complications has accelerated research into therapeutic strategies targeting senescent cell accumulation, including senolytics, which selectively eliminate these cells, and senomorphics, which modulate the SASP without directly ablating senescent cells [191]. Several senolytic agents, including the combination of dasatinib and quercetin, the natural flavonoid fisetin, and the BCL-XL inhibitor UBX1325, have entered phase I and II clinical trials for diabetes-related and age-associated conditions. In parallel, drugs already used to manage diabetes and related conditions, such as statins, metformin, SGLT2 inhibitors, and GLP-1 receptor agonists, have also shown potential senomorphic effects, suggesting additional therapeutic benefits by modulating cellular senescence. Early studies highlight significant improvements in insulin resistance, kidney function, and systemic inflammation by effectively clearing senescent cells or suppressing the senescence-associated secretory phenotype [191]. Furthermore, ongoing research into intermittent dosing regimens aims to optimize safety and therapeutic efficacy, potentially facilitating the development of senescence-targeting therapies for diabetes, its complications, and age-related diseases.

Excessive ROS production is a key driver of myocardial dysfunction, structural remodeling, and heart failure progression in diabetic cardiomyopathy. Consequently, emerging therapeutic approaches have shifted from non-targeted antioxidant scavenging to mechanism-based precision interventions that selectively modulate redox balance at primary cellular sources, such as mitochondria and NADPH oxidase. Looking ahead, future strategies focus on targeting redox-sensitive transcription factors and enzymes like Nrf2, NOX isoforms, and SIRT1 to restore endogenous redox homeostasis, as activating protective pathways or inhibiting ROS-generating sources mitigates myocardial injury. Furthermore, advanced delivery platforms—including nanotechnology and mitochondria-targeted peptides—offer novel avenues to overcome poor bioavailability and off-target limitations [192].

## 5. Conclusions

**Figure 6:**
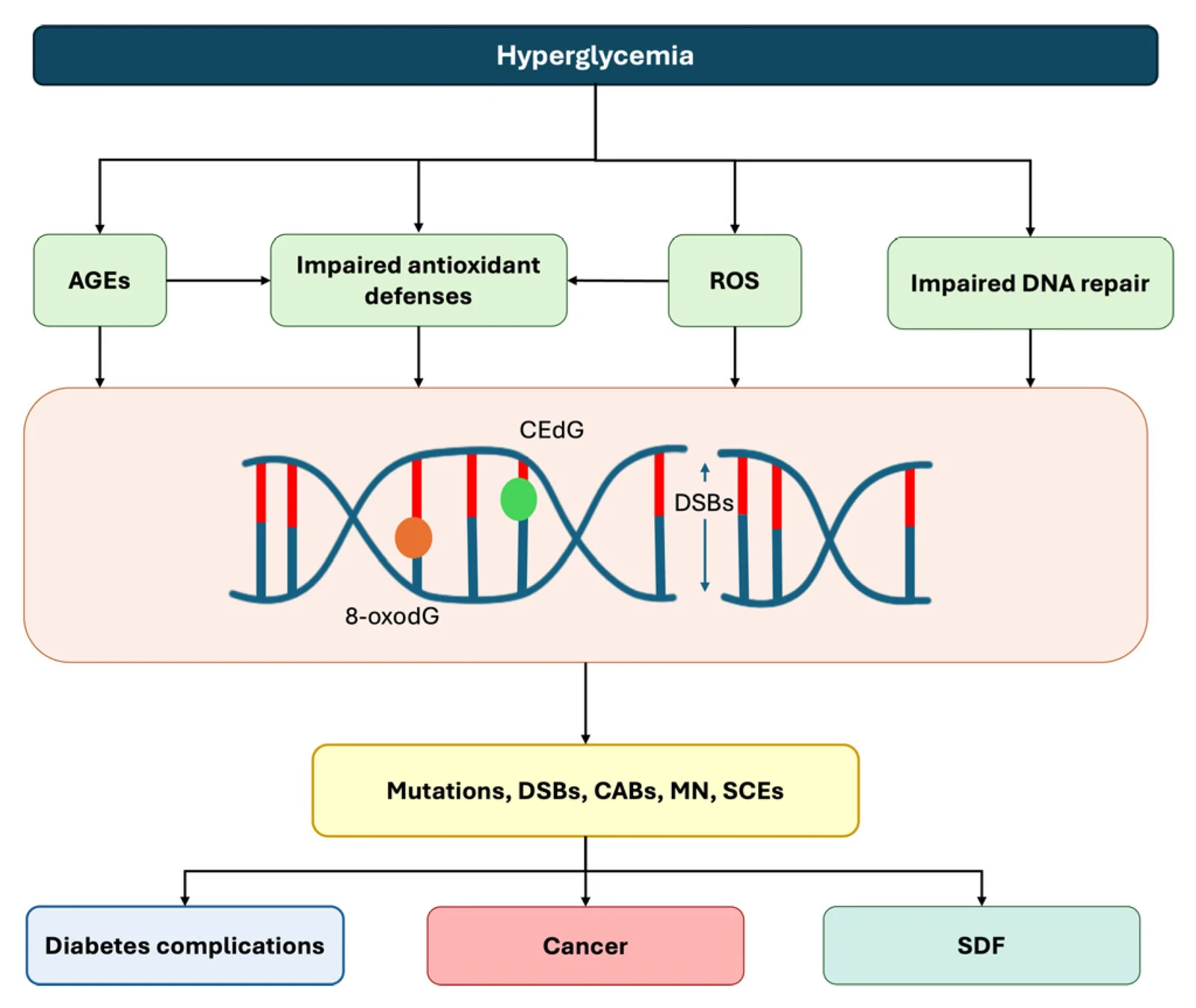
Hyperglycemia-induced DNA damage mechanisms and downstream pathological outcomes. Hyperglycemia causes genomic instability by simultaneously generating AGEs, elevating ROS, and impairing DNA repair. AGEs and ROS induce specific DNA lesions, including AGE-DNA adducts (CEdG), oxidative base modifications (8-oxo-dG), and double-strand breaks (DSBs), while both AGE accumulation and ROS contribute to impaired cellular antioxidant defenses. Reduced DNA repair efficiency further prevents the clearance of these lesions. Unrepaired DNA damage progresses to permanent genomic alterations, including mutations, DSBs, chromosomal aberrations (CABs), micronuclei (MN), and sister chromatid exchanges (SCEs). These structural defects ultimately drive diabetic complications, carcinogenesis, and sperm DNA fragmentation (SDF).

In conclusion, compelling evidence indicates that DNA damage represents a relevant pathological feature in diabetes. This damage is primarily driven by hyperglycemia, which promotes intracellular ROS formation through multiple pathways. Furthermore, AGEs contribute to impaired genomic integrity, both by forming direct DNA adducts and by stimulating further ROS production. When these lesions escape cellular repair, they may trigger cellular senescence, apoptosis, or create a pro-mutagenic environment that increases the risk of diabetes complications or cancer development. In addition, DNA damage contributes to related conditions such as male infertility through sperm DNA fragmentation (Figure 6). More recent research has highlighted that novel molecular cascades triggered by DNA damage—especially those arising from impaired DNA repair—can ultimately promote emerging forms of cell death, including ferroptosis and pyroptosis. Together with fibrosis, these processes contribute to the progressive organ dysfunction associated with diabetic complications. Consequently, preclinical models remain essential for elucidating these underlying mechanisms and identifying targetable pathways, with the ultimate aim of developing interventions that mitigate both systemic complications and cancer risk in diabetic patients.

## Data Availability

No new data were created or analyzed in this study. Data sharing is not applicable to this article.

## Abbreviations

The following abbreviations are used in this manuscript:

GAPDH	Glyceraldehyde 3-Phosphate Dehydrogenase
PARP-1	Poly (ADP-ribose) Polymerase 1
PKC	Protein Kinase C
NOX	NADPH Oxidase
MGO	Methylglyoxal
PLP	Pyridoxal 5′-phosphate
SCEs	Sister Chromatid Exchanges
MN	Micronuclei
SDF	Sperm DNA Fragmentation
CABs	Chromosomal Aberrations
GSDMD	Gasdermin D
